# Surgical Debulking Modifies Notch Signaling and May Improve Vismodegib Effectiveness for Locally Advanced Basal Cell Carcinoma

**DOI:** 10.1016/j.xjidi.2024.100288

**Published:** 2024-06-06

**Authors:** Natella Maglakelidze, Samantha L. Gettle, Amy L. Longenecker, Allison T. Vidimos, Elizabeth M. Billingsley, Ryan P. Hobbs, Charlene Lam

**Affiliations:** 1 Department of Dermatology, Penn State College of Medicine, Hershey, Pennsylvania, USA; 2 Department of Dermatology, Cleveland Clinic, Cleveland, Ohio, USA; 3 Department of Microbiology and Immunology, Penn State College of Medicine, Hershey, Pennsylvania, USA

**Keywords:** Basal cell carcinoma, Notch signaling, Smoothened inhibitors

## Abstract

Smoothened inhibitors, such as vismodegib, exhibit remarkable success in treating patients with locally advanced basal cell carcinoma (LaBCC). Yet, vismodegib efficacy is hindered by notable side effects, which often lead to treatment discontinuation and subsequent relapse in patients with LaBCC. Prolonged remission was previously reported in patients with LaBCCs who underwent surgical debulking before starting vismodegib. In this study, we enrolled 4 patients with LaBCC who underwent debulking followed by vismodegib therapy to assess their clinical outcomes and analyze the cutaneous molecular changes occurring as a result of surgical intervention. After LaBCC debulking, patients underwent a punch biopsy of residual basal cell carcinoma tissue 1 week later. RT-qPCR analysis of 24 Notch and Wnt signaling–associated genes revealed elevated *PTCH1*, *HEY2*, *LGR6*, *FZD2*, *LEF1*, *ALCAM*, and *RUNX1* expressions in follow-up biopsies compared with those in patient-matched debulked tissue. Immunoblot and immunostaining further confirmed elevated Notch signaling in follow-up biopsy tissue compared with that in patient-matched debulked tumor tissue. Patients 1, 3, and 4 displayed a clinical response to debulking followed by vismodegib, whereas patient 2 was lost to follow-up after debulking. These findings suggest that surgical manipulation of LaBCCs is correlated with molecular alterations in signaling pathways associated with cellular reprogramming.

## Introduction

Basal cell carcinoma (BCC) is the most common type of carcinoma worldwide, with its incidence steadily rising (Dika et al, 2020). BCCs can cause considerable morbidity and profoundly impact patients’ QOL by instigating local tissue destruction. Although early diagnosis and treatment through surgical and destructive modalities often lead to favorable outcomes in the vast majority of BCC cases (Dika et al, 2020), a subset of patients present with more formidable forms of BCC that may exhibit extensive tissue destruction, deep invasiveness, high recurrence rates, and repetitive treatment failures. These forms, collectively termed locally advanced BCCs (LaBCCs), are estimated to account for 1–10% of all BCCs (Dika et al, 2020; Niebel et al, 2020; Mohan et al, 2014). Large, exophytic LaBCC lesions, especially when located on the head and neck, prove symptomatic, disfiguring, and capable of destroying essential sensory organs. Attaining clear surgical margins can be a challenge in LaBCCs, whereas extensive surgeries bear significant functional and aesthetic consequences. Thus, the management of LaBCCs often requires a multidisciplinary, multimodality approach (Niebel et al, 2020).

The Sonic hedgehog (Shh) signaling pathway plays a central role in the pathogenesis of most BCCs, including LaBCCs (Niebel et al, 2020). Inactivating sequence variants in the tumor suppressor PTCH are implicated in 90% of sporadic BCCs (Dika et al, 2020). Loss-of-function variants in PTCH1 negate its inhibition of the Shh pathway, leading to increased GLI transcription and promotion of BCC development and progression (Dika et al, 2020). Given its pivotal role in BCC pathogenesis, the Shh signaling pathway emerged as a promising therapeutic target. Smoothened (SMO) inhibitors, such as vismodegib, have been employed for LaBCCs and metastatic BCCs since their Food and Drug Administration approval in 2012 (Axelson et al, 2013). Nonetheless, their precise roles and treatment parameters, including treatment duration, remain unclear and vary among physicians. Furthermore, discontinuation of SMO inhibitors occurs in 40–80% of patients owing to notable side effects from prolonged use, disease progression, or mortality (Basset-Séguin et al, 2017; Sekulic et al, 2017; Slowinska et al, 2022). Although discontinuation rates may be mitigated through dose adjustments and managing the side effects (eg, calcium channel blockers to prevent muscle spasms, nutritional counseling to combat weight loss), these limitations dampen enthusiasm for SMO inhibitor use in treating LaBCCs long term. Moreover, the mechanisms underlying BCC regression remain incompletely understood, with residual tumor cells often persisting despite SMO inhibition, leading to BCC recurrence post-treatment discontinuation (Atwood et al, 2014).

Positive long-term outcomes, such as potentially reduced treatment duration and decreased tumor recurrence, were previously reported in 2 patients who underwent surgical debulking of large exophytic LaBCCs before commencing vismodegib treatment (Wirth et al, 2021). Injury-induced tissue regeneration has been previously shown to trigger cellular reprogramming, including the upregulation of Notch and Wnt signaling (Chen et al, 2017, 2012; Okamoto et al, 2009; Yanger et al, 2013). Previous studies of human BCC and mouse models for BCC demonstrated the compartmentalization of Notch and Wnt signaling within nodular BCCs that facilitated tumor persistence and recurrence, whereby Notch-positive cells were more susceptible to apoptosis and Wnt-activated cells were more prone to persist after vismodegib treatment (Biehs et al, 2018; Eberl et al, 2018; Sánchez-Danés et al, 2018; Shi et al, 2017; Zhao et al, 2015).

In this study, we aimed to ascertain whether surgical debulking of LaBCCs leads to enhancement in Notch and Wnt signaling within residual BCC tissue. We enrolled 4 patients with large, exophytic LaBCCs that were suitable for vismodegib therapy. Patients underwent an initial debulking of the exophytic portion of the tumor and then a punch biopsy 1 week after debulking prior to initiating vismodegib treatment. We investigated the surgical debulk–induced alterations in Notch and Wnt signaling and qualitatively correlated these changes with long-term patient outcomes.

## Results

### Clinical findings and outcomes of patients treated with vismodegib after surgical debulking of exophytic BCCs

**Figure 1:**
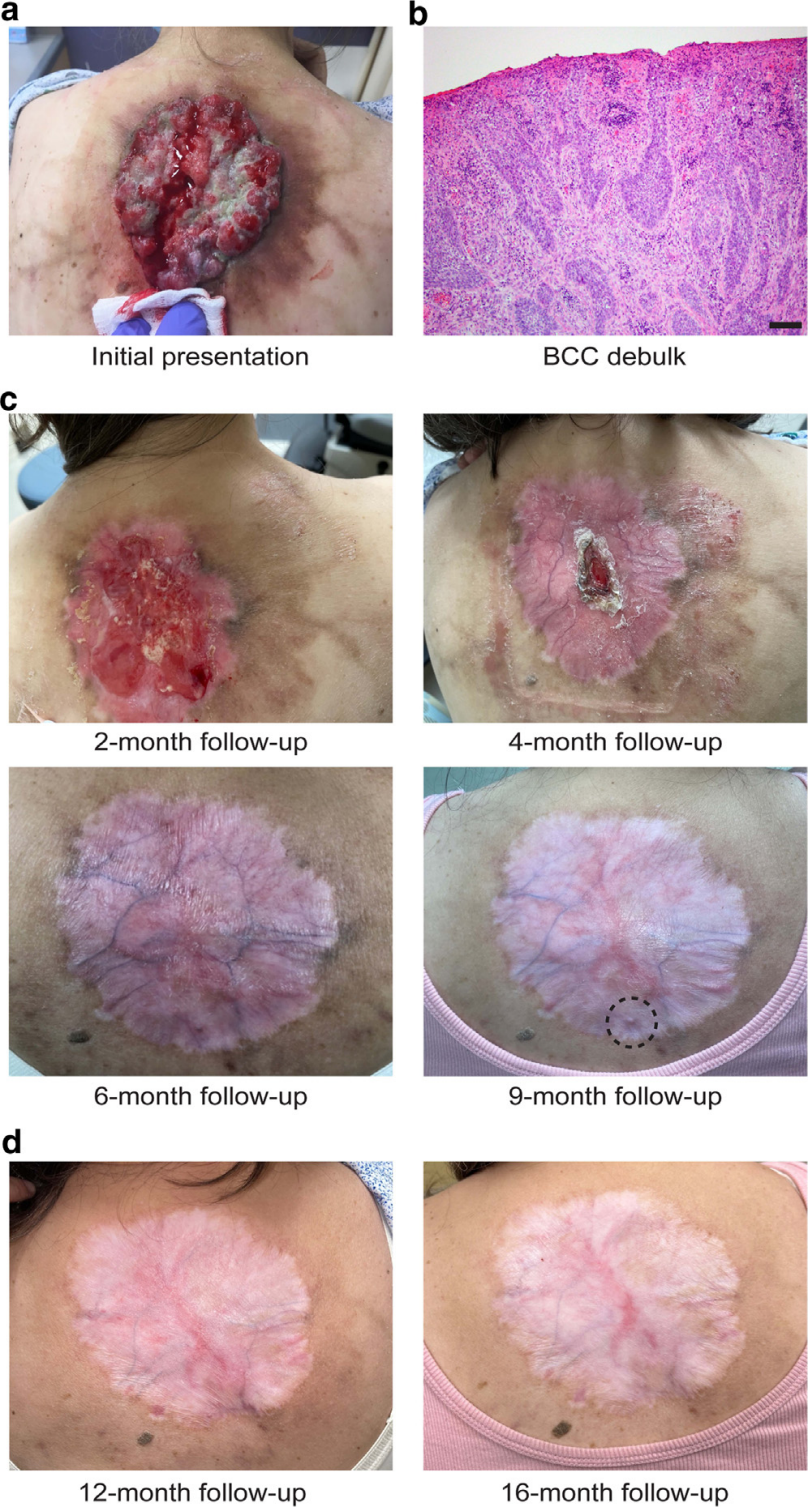
**Surgical debulking of exophytic BCCs prior to starting vismodegib therapy leads to positive long-term patient outcomes.** (**a**) Macroscopic image of exophytic BCC measuring 9.0 × 7.0 cm on central back (patient 4). **(b)** H&E staining of BCC debulk tissue at ×10 magnification (bar = 100 mm). **(c)** Macroscopic image of central back at 2-month, 4-month, 6-month, and 9-month follow-up after initiation of vismodegib therapy. **(d)** Macroscopic image of central back at 12- and 16-month follow-up after surgical excision of BCC recurrence near original tumor margin (dotted circle in 9-month photo). BCC, basal cell carcinoma.

Four patients with large, exophytic LaBCCs were recruited for this pilot study (Table 1). Patients 1, 2, and 3 presented with facial LaBCCs. Patient 2 had an additional LaBCC located on the chest. Patient 4 presented with a LaBCC on the central back (Figure 1a). All 4 patients had a standard-of-care biopsy performed at each site, and a definitive histologic diagnosis of BCC was made (Figure 1b). Each patient then underwent an initial debulk of the exophytic portion of the tumor. One week later, a 4-mm follow-up punch biopsy was obtained from the residual BCC tissue. Patients 1, 3, and 4 were started on a standard course of vismodegib therapy (150 mg taken orally daily). Patient 2 was lost to follow-up and not started on vismodegib. Duration of vismodegib therapy varied per patient (7.5, 9, and 5 months) with an average of 7.2 months (Table 1). Patient 1 continued to exhibit no signs of tumor recurrence 20 months after cessation of vismodegib. Patient 3 exhibited an initial complete clinical and histologic response to vismodegib, leading to its discontinuation. However, 4 months after their last vismodegib treatment, a subcutaneous preauricular nodule was identified as a BCC. During Mohs micrographic surgery for this lesion, a separate and distinct BCC was discovered inferior to the primary preauricular site. Given the depth and extent of involvement by these 2 BCC lesions, it was hypothesized that it may be extending from the original postauricular BCC. The patient ultimately required an auricectomy to achieve clear surgical margins because he did not want to go back on the vismodegib. Patient 4 developed a nodular BCC (3 mm) near the edge of the original BCC 4 months after vismodegib discontinuation and underwent surgical excision with 4 mm margins of the lesion (Figure 1c). Patient 4 has no signs of tumor recurrence 12 months after the surgical excision of the nodular BCC and 16 months after vismodegib discontinuation (Figure 1d).

**Table 1:** Clinical Findings and Outcomes of Patients Treated with Vismodegib after Surgical Debulking of LaBCCs

Patient	Sex	Age (y)	Tumor Location	Tumor Size	Duration of Vismodegib after Debulk	Patient Outcomes
1	Male	86	Right posterior ear	5.5 × 3.5 cm	7.5 mo	No BCC recurrence Remission, 20 months after last vismodegib dose
2	Male	62	a. Right forehead b. Left chest	a. 4.5 × 4.1 cm b. 6.3 × 5.6 cm	N/A	Lost to follow-up after tumor debulk and follow-up biopsy
3	Male	79	Left posterior ear	6.0 × 5.5 cm	9 mo	No BCC recurrence at original site Biopsy-proven BCC on the right preauricular cheek, 4 mo after the last vismodegib dose
4	Female	65	Central back	9.0 × 7.0 cm	5 mo	Biopsy-proven nodular BCC recurrence near original tumor margin, 3 mo after the last vismodegib dose No BCC recurrence 12 mo after surgical excision

Abbreviations: BCC, basal cell carcinoma; LaBCC, locally advanced basal cell carcinoma; N/A, not available.

### Surgical debulking of exophytic BCCs leads to elevated expression of Notch- and Wnt-associated genes

**Figure 2:**
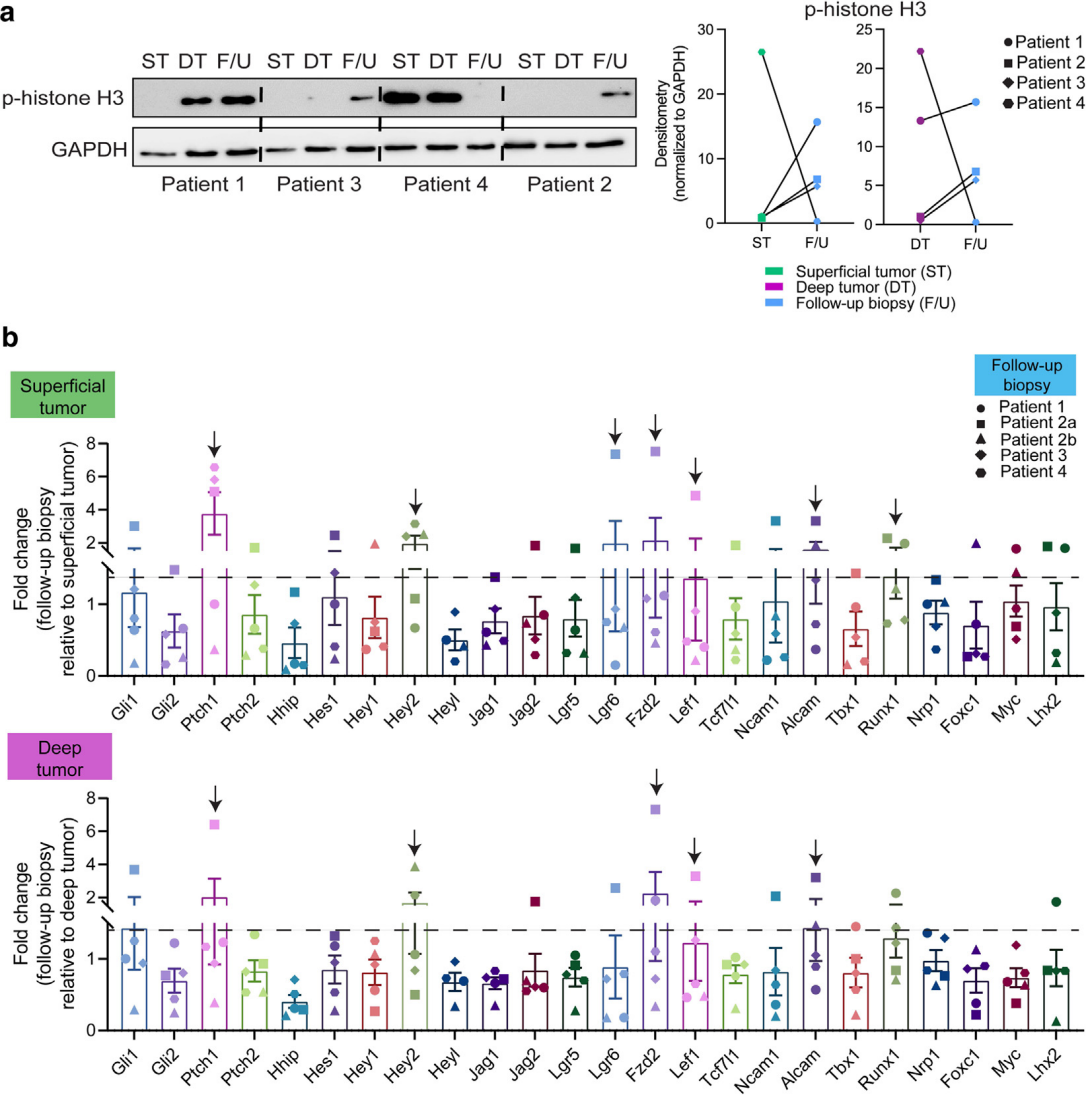
**Surgical debulking of exophytic BCCs leads to elevated expression of Notch- and Wnt-associated genes.** (**a**) Immunoblot analysis of phosphorylated H3 expression in ST, DT, and F/U biopsy for patients 1, 3, 4, and 2 (forehead only). Densitometry paired dot plot graphs are for the blot represented. All protein levels were normalized to GAPDH. **(b)** Expression of 24 Wnt and Notch signaling genes in F/U biopsy relative to patient-matched superficial (top) and deep (bottom) tumor debulk tissue. Dashed black line marks >1.5-fold change of *PTCH1* (HH), *HEY2* (Notch), *LGR6* (Wnt), *FZD2* (Notch), *LEF1* (Wnt), *ALCAM* (Wnt), and *RUNX1* (Notch/Wnt) compared with those of superficial and deep BCC debulk tissue as highlighted by black arrows. n = patient 1 (ear), patient 2a (forehead), patient 2b (chest), patient 3 (ear), and patient 4 (back). BCC, basal cell carcinoma; DT, deep tumor; F/U, follow-up; HH, hedgehog; ST, superficial tumor.

To assess the impact of surgical debulking on Notch and Wnt signaling pathways, we initially processed the patient follow-up biopsies alongside their matched debulked tissue samples for RT-qPCR, immunoblotting, and tissue embedding for cryosectioning and histologic analyses. Given the substantial size of the exophytic LaBCCs, each patient’s debulk sample was partitioned into 2 pieces to capture superficial and deep tumor tissue components. The superficial tumor tissue was more necrotic and ulcerated than the deep tumor tissue. Immunoblot analysis demonstrated higher phosphorylated histone H3 levels in the follow-up biopsies for 3 of the 4 patients (patient 1, patient 3, and patient 2 [forehead lesion]) than in their matched superficial and deep tumor tissue samples, suggesting increased proliferation likely due to wound healing and re-epithelialization occurring 1 week after surgical manipulation (Figure 2a). RT-qPCR analysis revealed that the follow-up biopsies had elevated expression (>1.5-fold change) of *PTCH1* (hedgehog), *HEY2* (Notch), *LGR6* (Wnt), *FZD2* (Notch), *LEF1* (Wnt), *ALCAM* (Wnt), and *RUNX1* (Notch/Wnt) compared with superficial and deep BCC debulk tissue (Figure 2b).

### NOTCH1 intracellular domain and HEY2 protein levels are abundant after surgical debulking of exophytic BCCs

**Figure 3:**
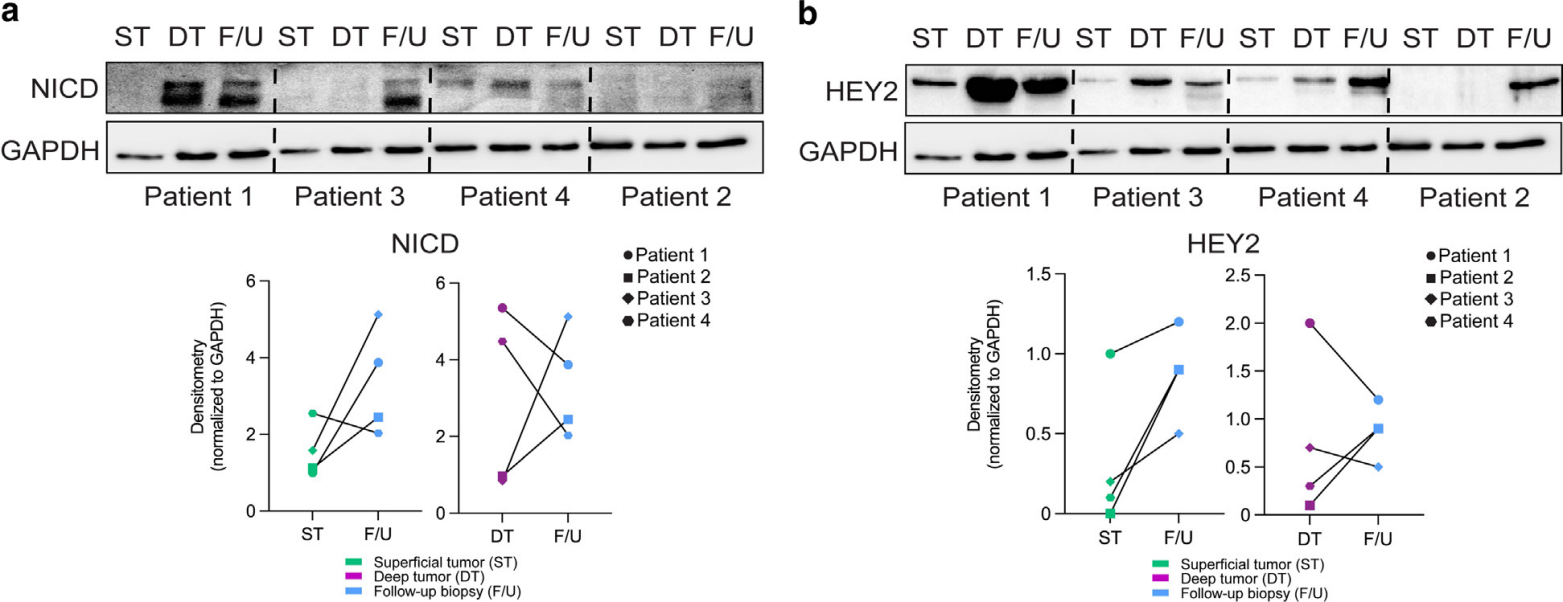
**NICD and HEY2 protein levels are elevated after surgical debulking of exophytic BCCs.** Immunoblot analysis of **(a)** NICD and **(b)** HEY2 expressions in ST, DT, and F/U biopsy for patients 1, 3, 4, and 2 (forehead only). Densitometry paired dot plot graphs are for the blots represented. All protein levels were normalized to GAPDH. BCC, basal cell carcinoma; DT, deep tumor; F/U, follow-up; NICD, NOTCH1 intracellular domain; ST, superficial tumor.

HEY2 is a transcription factor activated by NOTCH1 intracellular domain (NICD), which is the activated portion of the Notch receptor after stimulation (Okamoto et al, 2009). Because *HEY2* expression was elevated in the follow-up biopsies compared with that in BCC debulk tissue, we next assessed the protein levels of NICD and HEY2 in the superficial debulk tumor, deep debulk tumor, and follow-up biopsies. Immunoblot analysis revealed either elevated or persistent levels of NICD in the follow-up biopsies from all 4 patients compared with those in their matched superficial tumor tissues (Figure 3a). Similarly, immunoblot analysis of HEY2 demonstrated elevated or persistent levels of HEY2 in the follow-up biopsies from all 4 patients compared with those in their matched superficial tumor tissue (Figure 3b).

**Figure 4:**
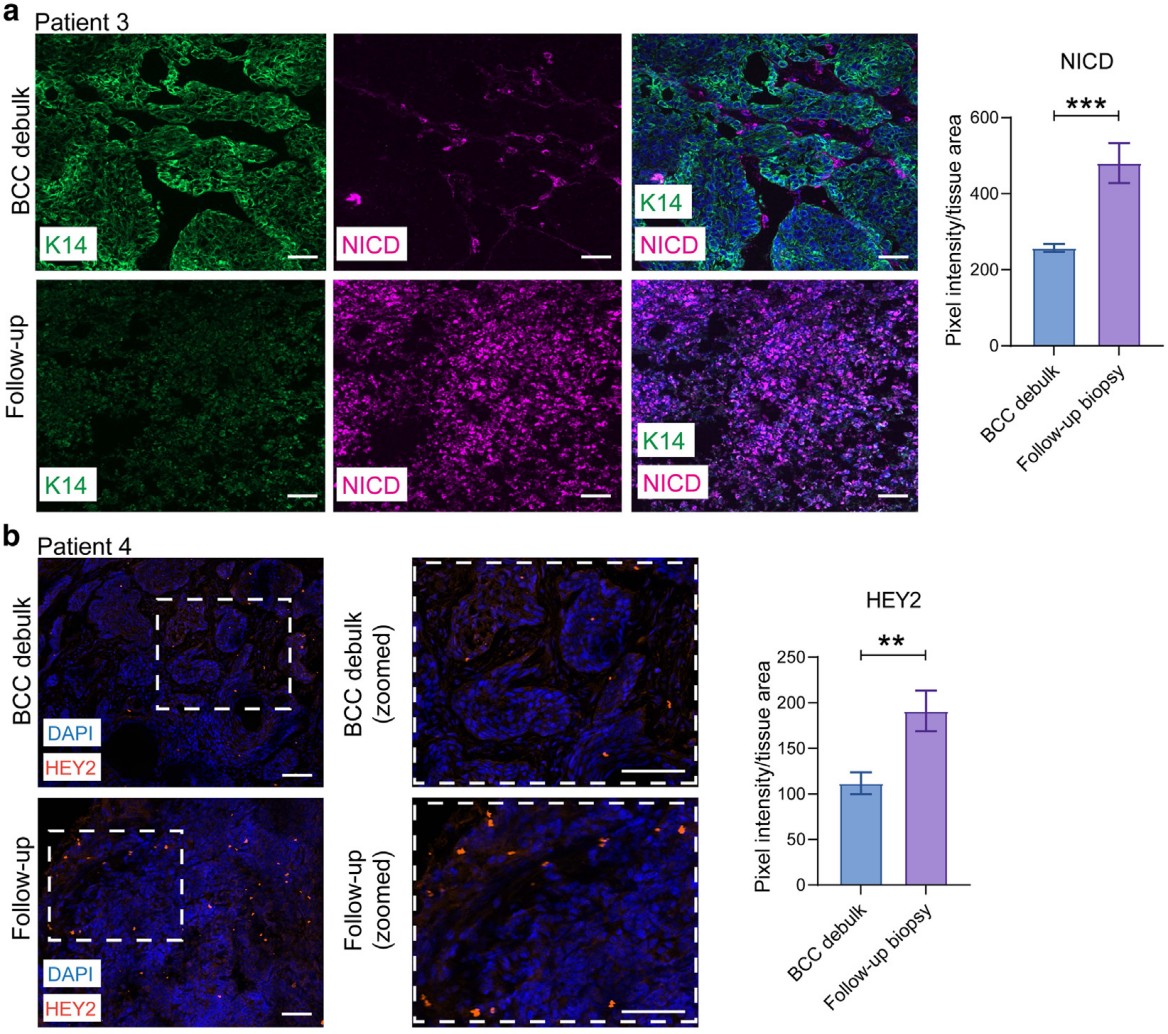
**NICD and HEY2 expressions are elevated after surgical debulking of exophytic BCCs.** (**a**) K14 (green) and NICD (pink) immunostaining of BCC debulk and patient-matched follow-up biopsy. Quantitation for patient 3 NICD expression (pixel intensity/mm^2^) is on the right. **(b)** HEY2 (orange) immunostaining of BCC debulk and patient-matched follow-up biopsy. Quantitation for patient 4 HEY2 expression (pixel intensity/mm^2^) is on the right. DAPI = blue. Bar graphs represent mean pixel intensity/mm^2^ ± SEM. Bar = 20 μm. ∗∗*P* < .01 and ∗∗∗*P* < .001. BCC, basal cell carcinoma; K14, keratin 14; NICD, NOTCH1 intracellular domain.

To analyze the distribution of NICD and HEY2 expression within the BCC debulk and follow-up biopsy tissues, we conducted immunofluorescence staining of tissue cryosections. For NICD, we observed robust and widespread upregulation (+87%, *P* < .001) in the follow-up biopsy tissue compared with that in the BCC debulk tissue (Figure 4a). The BCC debulk tissue revealed minimal NICD expression in the basal and suprabasal tumor cells. The tumor architecture in the follow-up biopsy tissue was significantly disrupted after surgical manipulation, with no distinct basal or suprabasal cell compartments evident on immunostaining (Figure 4a). We also observed upregulation of HEY2 expression (+71%, *P* = .0071) in the follow-biopsy tissue compared with that in the BCC debulk tissue (Figure 4b). Similar to NICD, HEY2 did not localize to a particular basal or suprabasal tumor compartment. Overall, our findings suggest that surgical manipulation of exophytic BCC tissue disrupts tissue architecture and is correlated with elevated Notch signaling.

In summary, our study indicated that surgical debulking of exophytic BCCs prior to the initiation of vismodegib therapy was associated with a positive clinical response, with 2 of the 3 patients we were able to follow experiencing long-term remission after vismodegib discontinuation. This may be due to the persistent or elevated expression of Notch signaling in residual tumor cells after surgical manipulation, a phenomenon previously shown to render residual BCC tumor cells susceptible to vismodegib-induced apoptosis.

## Discussion

BCCs are a pervasive form of carcinoma, with treatment strategies contingent upon factors such as tumor size, depth, and location. Targeted systemic therapy using SMO inhibitors, notably vismodegib, has demonstrated impressive efficacy in treating large exophytic BCCs (Niebel et al, 2020). However, frequent tumor recurrence upon drug discontinuation, coupled with often intolerable side effects of long-term SMO inhibitor use, presents a significant therapeutic challenge (Basset-Séguin et al, 2017; Bertrand et al, 2021). In this pilot study, we investigated the clinical advantages of surgical debulking preceding the initiation of vismodegib treatment and qualitatively correlated these findings with the molecular aspects of surgically manipulated large exophytic LaBCCs. Clinically, we were able to follow 3 patients in our study for more than 12 months and observed favorable outcomes in 2 patients compared with those of current care standards for LaBCCs. Although vismodegib initially achieved a clinical and histologic response in patient 3, surgical intervention eventually became necessary. This observation raises important considerations regarding tumor extent and the adequacy of initial assessment. The clinical appearance and superficial biopsy findings may have inadequately represented the deep invasion of the tumor, contributing to the premature cessation of vismodegib therapy. It remains uncertain whether continued or reinitiated vismodegib treatment could have induced complete tumor resolution or further response. However, the patient elected to proceed with auriculectomy. Regardless, the molecular studies still support an upregulation of the Notch signaling pathway with surgical manipulation.

Notch and Wnt signaling pathways have been previously implicated in BCC recurrence (Notch) and persistence (Wnt) in human BCC and mouse models of BCC (Biehs et al, 2018; Eberl et al, 2018; Sánchez-Danés et al, 2018; Shi et al, 2017). We observed sustained levels of Notch signaling markers in follow-up biopsies from all 4 patients who underwent surgical debulking before starting vismodegib therapy. Among the Notch- and Wnt-associated genes analyzed, *HEY2* expression emerged as one of the most elevated in the follow-up biopsies compared with that in BCC debulk tissue. This suggests that surgical manipulation may enhance Notch signaling in the skin, which may explain the lengthy sustained remission observed in our patients receiving vismodegib therapy when coupled with previous findings of Notch signaling promoting vismodegib-induced apoptosis of residual BCC tumor cells. Upregulation of Notch signaling after surgical manipulation has been reported in other types of cancer as well (Chen et al, 2017; Okamoto et al, 2009; Okamoto et al, 2009). Collectively, these studies lend support to a molecular-based explanation for the benefits of surgical debulking before initiating SMO inhibitor therapy. Although increased bioavailability of vismodegib due to surgical debulking and increased angiogenesis may contribute to better outcomes, we believe that this is a less likely explanation for our clinical observations. Previous studies have shown that patients with smaller BCCs can also develop resistance to vismodegib therapy, suggesting that tumor size reduction alone does not necessarily improve outcomes or decrease recurrence rates (Sinx et al, 2018).

There are several limitations to this study. We enrolled a small number of patients, which is reflective of the rarity of large, exophytic LaBCCs. The low sample size precluded robust statistical analysis. Moreover, patient 2 did not receive vismodegib therapy and was lost to follow-up, leading to an inability to correlate this patient’s molecular analyses to their clinical outcome. Future studies with larger patient cohorts are necessary to validate the efficacy of surgical manipulation prior to SMO inhibition as a therapeutic approach for LaBCCs. Head-to-head clinical trials are warranted to ascertain whether surgical debulking enhances the long-term outcomes of patients with LaBCCs undergoing vismodegib therapy. Further exploration into the molecular mechanisms underlying the elevation of Notch signaling postsurgical manipulation and its direct impact on the response to vismodegib therapy is necessary. In conclusion, our study suggests that surgical debulking before initiating vismodegib therapy may have significant benefits for patients with large, exophytic LaBCCs. By potentially increasing the tumor’s susceptibility to SMO inhibitors, this combinatorial treatment strategy could reduce drug duration, minimize side effects, and decrease tumor recurrence.

## Materials and Methods

### Patient recruitment, institutional review board approval, and criteria

A total of 4 patients were recruited from our institution’s dermatology clinic. Eligible patients included those aged >18 years; those with large exophytic, LaBCCs with definitive histological diagnosis; and those who were going to be treated with an SMO inhibitor. Patients with metastatic BCCs were excluded. Certain histological subtypes were excluded, including basosquamous cell carcinoma and basal cell carcinosarcoma. Each patient had the tumor debulked, and the tumor was divided for formalin-fixed permanent pathology and RNAlater solution. With a definitive BCC histologic diagnosis, the patient returned to the clinic in 1 week, and a 4-mm punch biopsy was performed at the site where the BCC was debulked and placed in RNAlater solution. An SMO inhibitor was then initiated as per standard of care. The study was approved by our institution’s Institutional Review Board (00013885).

### Tissue biopsy and morphological analyses

Exophytic BCC tissues and follow-up biopsies were obtained from patients and stored in RNALater at −20 ⁰C. Each sample was grossly dissected in half (half for RNA and protein isolation and half for tissue sectioning and staining). For RNA and protein isolation, BCC debulk tissue was further dissected in half to separate superficial tumor from deep tumor tissue. For staining, all tissue samples were embedded in cryomolds using optimal cutting temperature compound. Vertical and horizontal tissue cross-sections of 7 μm were generated using a cryostat (CM1850, Leica). Tissues were stained with H&E using a standard histology protocol (Hobbs et al, 2015).

### RT-qPCR analyses

RT-qPCR work followed MIQE (Minimum Information for Publication of Quantitative Real-Time PCR Experiments) guidelines. Exophytic BCC tissues and follow-up tissues were isolated and stored in RNALater at −20 ⁰C. RNA was isolated using Trizol (Invitrogen), followed by DNAseI treatment (Macherey-Nagel) and NucleoSpin RNA Mini column cleanup (Macherey-Nagel). RNA concentration and purity were assessed by spectrophotometry. A total of 1 μg of RNA was reverse transcribed using iScript (Bio-Rad Laboratories) or qScript (Quanta Bio). RT-qPCR was performed using PerfeCta SYBR Green (Quanta Bio) and Light Cycler 480 (Roche). Parameters include 95 °C for 3 minutes, 45 cycles of 95 °C for 5 seconds, and 60 °C for 30 seconds. Data analysis was performed using LightCycler 96 (Roche). Normalized expression was calculated using the 2^-DDCq^ method in Microsoft Excel by averaging the relative expression for each target gene (2^[CqTarget-CqReference]^ = 2^DCq^) across all biological replicates and dividing the average relative expression of the experimental condition by the control condition (2^Δ^^CqExperimental^ / 2^Δ^^CqControl^ = 2^-^^ΔΔ^^Cq^). Normalized expression for each target gene was determined from at least 3 biological replicates. Error bars represent the SEM of fold change values. *ACTB*, *GAPDH*, and *RPS18* were used as reference genes. The same reference genes were used for all PCR reactions, and all PCRs were normalized to all 3 reference genes. Owing to the limited patient sample size, we lacked sufficient power to perform statistical analyses.

### Western blotting analysis and imaging

Protein was isolated from exophytic BCC tissues and follow-up biopsies using Trizol (Invitrogen), solubilized in 1× SDS sample buffer, and boiled for 10 minutes. Protein samples were resolved by SDS-PAGE and transferred onto a polyvinylidene difluoride membrane. Membranes were probed using rabbit polyclonal antibodies against cleaved NOTCH1 (NICD) (110 kDa), HEY2 (36 kDa), and phosphorylated histone H3 (17 kDa). GAPDH (36 kDa) was used as a loading control. Antibody dilutions are provided in Table 2, Table 3, Table 4, Table 5, Table 6. SignalFire Plus ECL Reagent (Cell Signaling Technology) was used per the manufacturer’s instructions to detect protein levels using a Chemi-Doc MP Imaging System (Bio-Rad Laboratories). Comparisons of protein levels were quantified using ImageJ. Protein levels were normalized within the same gel. Owing to the limited patient sample size, we lacked sufficient power to perform statistical analyses.

**Table 2:** Primary Antibodies Used in this Study for Immunostaining (IF)/Immunoblotting (WB)

Human Primary Antibody	Company	Species	Clone	Catalog Number	Dilution
Cleaved Notch1 (Val1744)	Cell Signaling Technology	Rabbit	D3B8	4147	1:100 (IF) 1:1000 (WB)
HEY2	GeneTex	Rabbit		GTX128029	1:50 (IF) 1:500 (WB)
Keratin 14	BioLegend	Chicken	Poly9060	906004	1:100 (IF)
Phosphorylated histone H3 (Ser10)	Cell Signaling Technology	Rabbit		9701	1:500 (WB)
GAPDH	ProteinTech	Rabbit		10494-1-AP	1:10,000 (WB)

Abbreviations: IF, immunofluorescence; WB, western blotting.

**Table 3:** Secondary Antibodies Used in this Study for Immunostaining (IF)/Immunoblotting (WB)

Human Secondary Antibody	Company	Species	Catalog Number	Dilution
Anti-rabbit IgG, HRP-linked	Cell Signaling Technology	Rabbit	7074P2	1:2000 (WB)
Goat anti-rabbit IgG (H+L) highly cross-adsorbed secondary antibody, Alexa Flour 647	Thermo Fisher Scientific	Rabbit	A-21245	1:1000 (IF)
Goat anti-chicken IgY (H+L) cross-adsorbed secondary antibody, Alexa Fluor Plus 488	Thermo Fisher Scientific	Chicken	A32931	1:1000 (IF)

Abbreviations: HRP, horseradish peroxidase; IF, immunofluorescence; WB, western blotting.

**Table 4:** The IgG Control Used in this Study

IgG Controls	Company	Species	Catalog Number
Rabbit IgG	Cell Signaling Technology	Rabbit	3900S

IgG control dilutions/concentrations were matched to the primary antibody used.

**Table 5:** Reagents

Reagent	Company	Catalog Number	Dilution
BSA	Fisher bioreagents	BP9703-100	1%
DAPI	Thermo Fisher Scientific	62247	1:1000
Goat serum	Sigma-Aldrich	G9023	5%

**Table 6:** Primers

Gene Name	Species	Forward/Reverse	Sequence (5'–3')
*GLI1*	Human	Reverse	CAGCATGTACTGGGCTTTGAA
*GLI2*	Human	Forward	CTGCCTCCGAGAAGCAAGAAG
*GLI2*	Human	Reverse	GCATGGAATGGTGGCAAGAG
*PTCH1*	Human	Forward	GAAGAAGGTGCTAATGTCCTGAC
*PTCH1*	Human	Reverse	GTCCCAGACTGTAATTTCGCC
*PTCH2*	Human	Forward	GCTTCGTGCTTACTTCCAGGG
*PTCH2*	Human	Reverse	CATGCGGAGACCTAATGCCA
*HHIP*	Human	Forward	TCTCAAAGCCTGTTCCACTCA
*HHIP*	Human	Reverse	GCCTCGGCAAGTGTAAAAGAA
*HES1*	Human	Forward	TCAACACGACACCGGATAAAC
*HES1*	Human	Reverse	GCCGCGAGCTATCTTTCTTCA
*HEY1*	Human	Forward	GTTCGGCTCTAGGTTCCATGT
*HEY1*	Human	Reverse	CGTCGGCGCTTCTCAATTATTC
*HEY2*	Human	Forward	AAGGCGTCGGGATCGGATAA
*HEY2*	Human	Reverse	AGAGCGTGTGCGTCAAAGTAG
*HEYL*	Human	Forward	GGAAGAAACGCAGAGGGATCA
*HEYL*	Human	Reverse	CAAGCGTCGCAATTCAGAAAG
*JAG1*	Human	Forward	GTCCATGCAGAACGTGAACG
*JAG1*	Human	Reverse	GCGGGACTGATACTCCTTGA
*JAG2*	Human	Forward	TGGGCGGCAACTCCTTCTA
*JAG2*	Human	Reverse	GCCTCCACGATGAGGGTAAA
*LGR5*	Human	Forward	CTCCCAGGTCTGGTGTGTTG
*LGR5*	Human	Reverse	GAGGTCTAGGTAGGAGGTGAAG
*LGR6*	Human	Forward	TGGGGAACCCTCTGCTACAG
*LGR6*	Human	Reverse	CAGGTACTGGAATGCCGATCT
*FZD2*	Human	Forward	GTGCCATCCTATCTCAGCTACA
*FZD2*	Human	Reverse	CTGCATGTCTACCAAGTACGTG
*LEF1*	Human	Forward	AGAACACCCCGATGACGGA
*LEF1*	Human	Reverse	GGCATCATTATGTACCCGGAAT
*TCF7L1*	Human	Forward	TCGTCCCTGGTCAACGAGT
*TCF7L1*	Human	Reverse	ACTTCGGCGAAATAGTCCCG
*NCAM1*	Human	Forward	GGCATTTACAAGTGTGTGGTTAC
*NCAM1*	Human	Reverse	TTGGCGCATTCTTGAACATGA
*ALCAM*	Human	Forward	TCCTGCCGTCTGCTCTTCT
*ALCAM*	Human	Reverse	TTCTGAGGTACGTCAAGTCGG
*TBX1*	Human	Forward	ACGACAACGGCCACATTATTC
*TBX1*	Human	Reverse	CCTCGGCATATTTCTCGCTATCT
*RUNX1*	Human	Forward	CTGCCCATCGCTTTCAAGGT
*RUNX1*	Human	Reverse	GCCGAGTAGTTTTCATCATTGCC
*NRP2*	Human	Forward	GCTGGCTATATCACCTCTCCC
*NRP2*	Human	Reverse	TCTCGATTTCAAAGTGAGGGTTG
*FOXC1*	Human	Forward	GGCGAGCAGAGCTACTACC
*FOXC1*	Human	Reverse	TGCGAGTACACGCTCATGG
*MYC*	Human	Forward	GGCTCCTGGCAAAAGGTCA
*MYC*	Human	Reverse	CTGCGTAGTTGTGCTGATGT
*LHX2*	Human	Forward	ATGCTGTTCCACAGTCTGTCG
*LHX2*	Human	Reverse	GCATGGTCGTCTCGGTGTC
*RPS18*	Human	Forward	GCGGCGGAAAATAGCCTTTG
*RPS18*	Human	Reverse	GATCACACGTTCCACCTCATC
*GAPDH*	Human	Forward	AAGGTGAAGGTCGGAGTCAAC
*GAPDH*	Human	Reverse	GGGGTCATTGATGGCAACAATA
*ACTB*	Human	Forward	CATGTACGTTGCTATCCAGGC
*ACTB*	Human	Reverse	CTCCTTAATGTCACGCACGAT

### Immunofluorescence microscopy and imaging

Frozen 7-μm exophytic BCC tissues and follow-up tissue sections were air dried, blocked, and stained overnight with anti-human antibodies at 4 °C. Sections were blocked in 5% goat serum and 1% BSA for 1 hour at room temperature. PBS with Tween was used to wash stained sections. Sections were stained with DAPI (1:1000) for 10 minutes. Slides were mounted and cover slipped with Fluorosave. Images were acquired using a Zeiss AxioObserver microscope, version 7.0. Images from similar experiments were equally brightened, contrasted, and cropped using Zen and ImageJ. For some representative images, the contrast was changed globally within ImageJ, and matching settings were applied to test and control. Pixel intensity/mm^2^ was quantified using ImageJ. Three negative controls were used for every immunofluorescence experiment (primary antibody only, secondary antibody only, and IgG isotype control matched to primary antibody host species). Antibody dilutions and reagents are provided in Table 2, Table 3, Table 4, Table 5, Table 6.

### Data presentation and statistical analyses

All figures were assembled in Adobe Illustrator (2022). All graphs were plotted in Prism 8 (GraphPad). Microscopy data are presented as mean ± SEM and compared using paired 2-tailed Student’s *t*-tests. All statistical analyses were performed using Prism 8.

## Ethics Statement

Human subjects research in this study was approved by the Penn State Institutional Review Board (institutional review board approval number 00013885). Written, informed consent was obtained for all patients at the time of their enrollment in this study.

## Data Availability Statement

Datasets related to this article can be found at https://scholarsphere.psu.edu/resources/2ff99333-dafb-4ab7-ad04-68c2c7800be4.

## ORCIDs

Natella Maglakelidze: http://orcid.org/0000-0001-6441-4753

Samantha L. Gettle: http://orcid.org/0000-0002-5471-8670

Amy L. Longenecker: http://orcid.org/0000-0002-3804-2789

Allison T. Vidimos: http://orcid.org/0000-0002-2545-7985

Elizabeth M. Billingsley: http://orcid.org/0009-0006-9081-4180

Ryan P. Hobbs: http://orcid.org/0000-0002-2827-1828

Charlene Lam: http://orcid.org/0000-0001-9956-0822

## Conflict of Interest

CL served in an advisory role for the Clinical Council at Genentech. The remaining authors state no conflict of interest.

## References

[bib1] Atwood S.X., Whitson R.J., Oro A.E. (2014). Advanced treatment for basal cell carcinomas. Cold Spring Harb Perspect Med.

[bib2] Axelson M., Liu K., Jiang X., He K., Wang J., Zhao H. (2013). U.S. Food and Drug Administration approval: vismodegib for recurrent, locally advanced, or metastatic basal cell carcinoma. Clin Cancer Res.

[bib3] Basset-Séguin N., Hauschild A., Kunstfeld R., Grob J., Dréno B., Mortier L. (2017). Vismodegib in patients with advanced basal cell carcinoma: primary analysis of STEVIE, an international, open-label trial. Eur J Cancer.

[bib4] Bertrand N., Guerreschi P., Basset-Seguin N., Saiag P., Dupuy A., Dalac-Rat S. (2021). Vismodegib in neoadjuvant treatment of locally advanced basal cell carcinoma: first results of a multicenter, open-label, phase 2 trial (VISMONEO study): neoadjuvant vismodegib in locally advanced basal cell carcinoma. EClinicalMedicine.

[bib5] Biehs B., Dijkgraaf G.J.P., Piskol R., Alicke B., Boumahdi S., Peale F. (2018). A cell identity switch allows residual BCC to survive Hedgehog pathway inhibition. Nature.

[bib6] Chen C.Y., Chen Y.Y., Hsieh M.S., Ho C.C., Chen K.Y., Shih J.Y. (2017). Expression of Notch gene and its impact on survival of patients with resectable non-small cell lung cancer. J Cancer.

[bib7] Chen H.T., Cai Q.C., Zheng J.M., Man X.H., Jiang H., Song B. (2012). High expression of delta-like ligand 4 predicts poor prognosis after curative resection for pancreatic cancer. Ann Surg Oncol.

[bib8] Dika E., Scarfì F., Ferracin M., Broseghini E., Marcelli E., Bortolani B. (2020). Basal cell carcinoma: a comprehensive review. Int J Mol Sci.

[bib9] Eberl M., Mangelberger D., Swanson J.B., Verhaegen M.E., Harms P.W., Frohm M.L. (2018). Tumor architecture and Notch signaling modulate drug response in basal cell carcinoma. Cancer Cell.

[bib10] Hobbs R.P., DePianto D.J., Jacob J.T., Han M.C., Chung B.M., Batazzi A.S. (2015). Keratin-dependent regulation of Aire and gene expression in skin tumor keratinocytes. Nat Genet.

[bib11] Niebel D., Sirokay J., Hoffmann F., Fröhlich A., Bieber T., Landsberg J. (2020). Clinical management of locally advanced basal-cell carcinomas and future therapeutic directions. Dermatol Ther (Heidelb).

[bib12] Okamoto R., Tsuchiya K., Nemoto Y., Akiyama J., Nakamura T., Kanai T. (2009). Requirement of Notch activation during regeneration of the intestinal epithelia. Am J Physiol Gastrointest Liver Physiol.

[bib14] Sánchez-Danés A., Larsimont J.C., Liagre M., Muñoz-Couselo E., Lapouge G., Brisebarre A. (2018). A slow-cycling Lgr5 tumour population mediates basal cell carcinoma relapse after therapy. Nature.

[bib15] Sekulic A., Migden M.R., Basset-Seguin N., Garbe C., Gesierich A., Lao C.D. (2017). Long-term safety and efficacy of vismodegib in patients with advanced basal cell carcinoma: final update of the pivotal ERIVANCE BCC study. BMC Cancer.

[bib17] Shi F.T., Yu M., Zloty D., Bell R.H., Wang E., Akhoundsadegh N. (2017). Notch signaling is significantly suppressed in basal cell carcinomas and activation induces basal cell carcinoma cell apoptosis. Mol Med Rep.

[bib18] Wirth P.J., Hobbs R., Billingsley E., Vidimos A.T., Lam C. (2021). Sonic hedgehog pathway blockade after surgical debulk of large exophytic basal cell carcinomas. Dermatol Surg.

[bib20] Yanger K., Zong Y., Maggs L.R., Shapira S.N., Maddipati R., Aiello N.M. (2013). Robust cellular reprogramming occurs spontaneously during liver regeneration. Genes Dev.

[bib21] Zhao X., Ponomaryov T., Ornell K.J., Zhou P., Dabral S.K., Pak E. (2015). RAS/MAPK activation drives resistance to smo inhibition, metastasis, and tumor evolution in shh pathway-dependent tumors. Cancer Res.

